# Atypical Kaposi Sarcoma of the Tongue in HIV Positive Tanzanian Female

**DOI:** 10.1155/2015/851462

**Published:** 2015-11-17

**Authors:** Elichilia Shao, Flora Ruhangisa, Neema Minja, Kanankira Nnko, Denis Katundu, George Semango, Eva Mbwilo, Amos Mwasamwaja, Kajiru Kilonzo, Isaack Lyaruu

**Affiliations:** ^1^Department of Internal Medicine, Kilimanjaro Christian Medical Centre, P.O. Box 3010, Moshi, Tanzania; ^2^Kilimanjaro Christian Medical University College, Tumaini University Makumira, P.O. Box 2240, Moshi, Tanzania; ^3^Better Human Health Foundation, P.O. Box 1348, Moshi, Tanzania; ^4^Image Doctors International, P.O. Box 16341, Arusha, Tanzania; ^5^Department of Pathology, Kilimanjaro Christian Medical Centre, P.O. Box 3010, Moshi, Tanzania; ^6^Endoscopy Unit, Kilimanjaro Christian Medical Centre, P.O. Box 3010, Moshi, Tanzania

## Abstract

We report atypical case of Kaposi Sarcoma (KS) in a 32-year-old human immunodeficiency virus- (HIV-) infected female, involving only the tongue. Viral loads and CD4 T cells were measured and were 65,000 cps/mL and 10 cells/mL, respectively. This patient was newly diagnosed and had no history of antiretroviral therapy (ART), radiotherapy, or immunosuppressive drugs prior to this admission. Clinically, there were no dermatological features of KS lesions which are purple, red, or brown and which may be flat, raised (plaques), or bumpy (nodules) except for fungating and protruding enlarged tongue which was necrotic. Histologically, it was proven to be the most common type of KS “epidemic” or AIDS-related KS.

## 1. Introduction

Kaposi Sarcoma (KS) is the soft tissue cancer that develops from the cells that line lymph or blood vessels [1]. It is caused by infectious virus called Kaposi Sarcoma Associated Herpesvirus (KSHV), also named as human herpes virus type 8 (HHV8) [2]. The disease mostly affects mucocutaneous tissues and aerodigestive tract. Besides body cavity based lymphoma (primary effusion lymphoma), Castleman's disease has also been associated with HHV-8 [3, 4]. It has been categorized into four epidemiological types based on the underlying causes. It can be classified into epidemic (AIDS-related), classic (Mediterranean/Sporadic), endemic (African), and iatrogenic (transplant related). Recently, there is a new class of KS in HIV negative men who have sex with men which is mild and can be similar to classic KS. HHV-8 transmission among men having sex with men is related to sexual risk factors such as number of sexual partners and to sexual practices involving saliva [2–6].

Epidemic KS among HIV patients is most commonly reported in Africa since the 1980s seen in about 40% of all HIV/AIDS patients. It has been linked with other diseases like primary effusion lymphoma [4]. Its transmission route is similar to that of HIV/AIDS; it is believed that infection among less than 10-year-old children is resulting from mother to child infection [3]. Its pathogenesis is multifactorial, though HIV is not a direct factor but it provides favourable environment for opportunistic factors such as very low T lymphocytes cells and very high viral copies. Some of HIV accessory genes such as transactivating (tat) gene and cytokines including oncostatin M play a big role in the development of aggressive KS among AIDS patients [7, 8]. KS is a treatable disease when diagnosed at the early stage, it has been noted that ART alone improves the outcome of AIDS-related KS. Patients with aggressive KS can be treated with paclitaxel, bleomycin, and vinblastine or vincristine (ABV) [3, 9, 10].

## 2. Case Presentation

**Figure 1:**
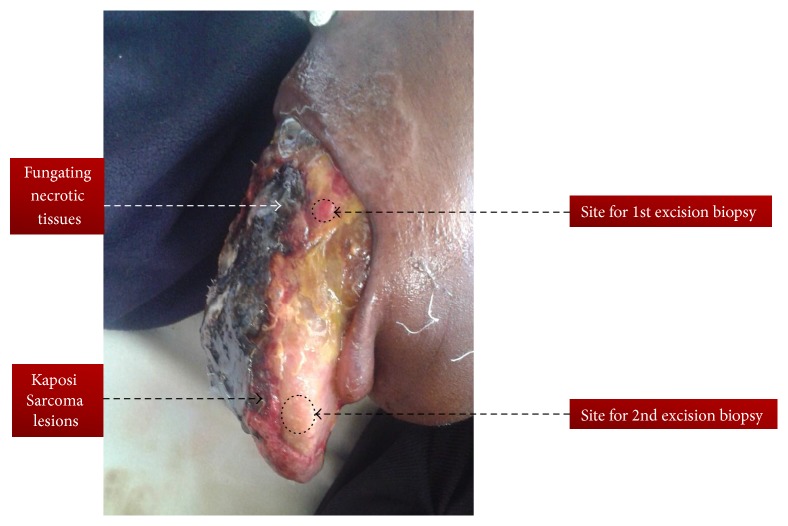
Enlarged and fungating tongue completely occluding oral cavity as a result of infiltrating Kaposi Sarcoma in HIV positive Maasai female.

**Figure 2:**
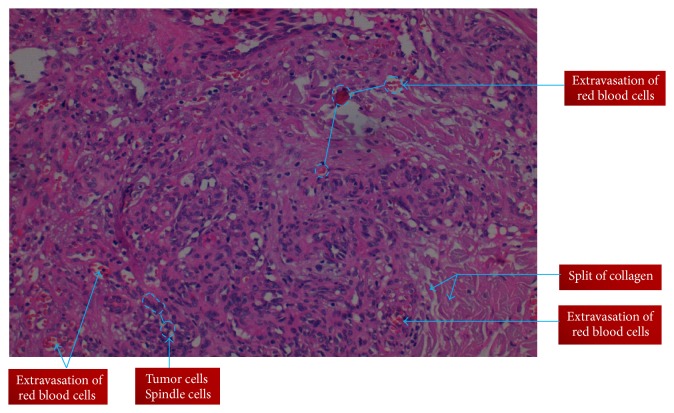
Histological features of Kaposi's sarcoma of the tongue in HIV positive Maasai female.

This was a thirty-two-year-old Maasai (one of pastoral tribes in Tanzania) female who was referred to our tertiary hospital in June 2015 with two weeks' history of recurrent fever, inability to swallow, weight loss, enlargement and protrusion of the tongue, and difficulty in speech. Her weight on time of admission was 43 kgs and height was 158 cm: Body Mass Index (BMI) was 17.22 Kg/m^2^. She was chronically ill with big necrotic and fungating protruding large tongue filling the whole oral cavity. The tongue has mixed lesions, that is, necrotic tissues and fresh wounds with easy bleeding plaques on the dorsal aspects of the tongue. She had also fungal infection (dermatophytes) of the upper lip (Figure 1). This tongue became large to the point that it occluded the oral cavity and made it difficult for her to speak or eat; it was accompanied by difficulty in breathing. Her tongue showed a cream coloured plaque at the lateral aspect with erythematous margin which was easily bleeding. Radiographic chest findings were remarkably normal; sputum microscopy for Acid Fast Bacilli was negative. Full blood picture was essentially normal except for the ESR which was 88 mm/hr, CD4 count 13 cells/*μ*L, and CD4% of 2.2. Abdominal ultrasound was performed and revealed no mass or organomegaly. Liver functional test revealed hypoalbuminemia with normal range enzymes. Serology for hepatitis B surface antigen (HBsAg) and hepatitis C were all negative. Our working diagnoses were World Health Organization (WHO) Clinical stage 4 HIV disease, septicaemia, oropharyngeal candidiasis, upper airway obstruction, and lingual carcinoma with malnutrition. She commenced first-line ART (Tenofovir, Lamivudine, and Efavirenz) alongside antibiotics Ampicillin and Metronidazole, Flucanzole, and oxygen therapy. On the fourth day in the ward, the condition of the patient changed and became dyspnoeic with oxygen saturation of 40% on oxygen therapy. Intubation was tried but was unsuccessful due to enlarged tongue occluding the oropharynx; tracheostomy was performed but patient condition deteriorated and she passed away. Histology results came out after one week and showed extravasations of red blood cells, collagen splitting, and spindle cells tumours (Figure 2).

## 3. Discussion

This case of histologically proven KS of the tongue in this patient with confirmed HIV infection illustrates that KS can present with only one feature of tongue enlargement. This was atypical presentation because there was no single lesion which was seen on the skin or from radiographic findings and ultrasonography. This was misdiagnosed as lingual carcinoma of the tongue or neurofibromatosis or fungal infection of the tongue. Routine tissue biopsy of the lesions among HIV positive patients is essential for the correct diagnosis. This case report had atypical presentation because it is very rare to see KS affecting only one organ because it has multiple sites. Skin is the most common site for KS to occur; it may involve the skin itself or the skin with other sites [11, 12]. In HIV era, burden of tuberculosis and* Pneumocystis jirovecii* pneumonia may mimic radiographic finding of pulmonary KS. Typical feature of pulmonary KS includes hilar lymphadenopathy, perihilar lymphadenopathy, lower zone infiltration, and effusions [13, 14]. In our case, the only positive findings were by histological results of excisional biopsy taken from the tongue in two different points with all concluding that it was KS.

Patients with KS need to be diagnosed and treated promptly to reduce mortality and morbidity. Atypical presentation of KS which involves only the tongue can happen and it is difficult to diagnose and treat. High index suspicion for KS in HIV infected individuals especially with CD4 count <200 cps/mL will be helpful in early diagnosis [11, 12]. Though we have few cases of KS reported in Tanzania, the current case was of interest due to its atypical clinical presentation [14]. Biopsy from the lesion and other sites which are easily accessible is crucial to exclude other opportunist conditions like lymphoma and tuberculosis.

## 4. Conclusions

We report the case of a 32-year-old female who died within 5 days since admission due to airway obstruction secondary to KS of the tongue confirmed histologically. She was initially suspected to have lingual carcinoma with upper airway obstruction. This case report underscores the importance of histology in diagnosing different types of malignancies as well as other opportunistic infections among HIV patients.
